# Major complications and mortality after ventral hernia repair: an eleven-year Swedish nationwide cohort study

**DOI:** 10.1186/s12893-022-01873-9

**Published:** 2022-12-13

**Authors:** Mikael Lindmark, Thyra Löwenmark, Karin Strigård, Ulf Gunnarsson

**Affiliations:** Department of Surgical and Perioperative Sciences, Umeå University, Umeå, Sweden

**Keywords:** Ventral hernia, Hernia repair, Risk factor, Complication, Mortality

## Abstract

**Background and aims:**

Ventral hernia repair is one of the most common surgical procedures performed worldwide. Despite the large volume, consensus is lacking regarding indications for repair or choice of surgical method used for reconstruction. The aim of this study was to explore the risk for major complications and mortality in ventral hernia repair using data from a nationwide patient register.

**Method:**

Patient data of individuals over 18 years of age who had a ventral hernia procedure between 2004 and 2014 were retrieved from the Patient Register kept by the Swedish National Board of Health and Welfare. After exclusion of patients with concomitant bowel surgery, 45 676 primary surgical admissions were included. Procedures were dichotomised into laparoscopic and open surgery, and stratified for primary and incisional hernias.

**Results:**

A total of 45 676 admissions were analysed. The material comprised 36% (16 670) incisional hernias and 64% (29 006) primary hernias. Women had a higher risk for reoperation during index admission after primary hernia repair (OR 1.84 (1.29–2.62)). Forty-three patients died of complications within 30 days of index surgery. Patients aged 80 years and older had a 2.5 times higher risk for a complication leading to reoperation, and a 12-fold higher mortality risk than patients aged 70–79 years.

**Conclusion:**

Age is the dominant mortality risk factor in ventral hernia repair. Laparoscopic surgery was associated with a lower risk for reoperation during index admission. Reoperation seems to be a valid outcome variable, while registration of complications is generally poor in this type of cohort.

## Introduction

Ventral hernia repair is one of the most commonly performed surgical procedures worldwide [1]. Despite the large numbers performed, the surgical community has difficulty in reaching consensus on the best approach for ventral hernia repair [2]. A large proportion of ventral hernias are suitable for repair in ambulatory surgery, but there are many complicated cases with considerable morbidity and even mortality [3]. Comparisons between patient series are questionable due to the heterogeneity of cohorts, and results are difficult to generalise. The current trend in management is an approach tailored to the individual’s requirements [4].

Many reported series do not differentiate between primary and incisional hernia. In series where these two are differentiated, significant differences frequently appear. Patients with incisional hernia are generally older, have higher BMI, higher ASA class, larger hernia, and more often suffer from type 2 diabetes [5]. A primary hernia may be regarded as a congenital condition due to innate weakness of the abdominal wall, while an incisional hernia is the result of defective wound healing or technical failure [5].

A systematic review of the literature and meta-analysis was published in 2019, which supports the hypothesis that primary ventral hernias and incisional hernias are different conditions with the latter being more challenging to treat. Their conclusion was that pooling data of primary and incisional hernias should no longer be performed in clinical trials [6].

Recurrence is traditionally the key outcome when assessing the quality of any form of hernia repair. In recent decades, recurrence rates have fallen due to the introduction of synthetic mesh for reinforcement. However, the use of synthetic mesh has not completely solved the problem of recurrence. Indeed, the current reoperation rate of 12.3% within five years [7] is high considering that actual recurrence rate can be four to five times higher than the reoperation rate [8]. Mesh repair has also introduced prosthesis-related side effects such as bowel obstruction, chronic site infection, and fistula formation. These are complications that partly offset the benefits of a lower recurrence rate, compared to suture repair [9].

Minimally invasive techniques have short-term advantages regarding surgical site complications [10]. Comparisons of laparoscopic intraperitoneal onlay mesh (IPOM) repair and open sublay mesh repair have shown similar outcomes for variables such as pain and time to full recovery [11]. Furthermore, there are reports of higher serious complication rates, including bowel injury, adhesions, and fistulae after laparoscopic repair [10]. Mandatory access to the abdominal cavity during IPOM repair is probably a contributing factor to these complications.

Recent years have seen rapid changes in trends and developments in hernia repair, and the future of ventral hernia management is difficult to foresee.

Hernia repair is a worldwide concern where the majority of procedures are not performed by dedicated hernia surgeons in highly specialised centres with access to the latest techniques. Thus, reports on cohorts with mainly open repairs are still of interest to the surgical community at large.

In Sweden, adoption of the laparoscopic approach for ventral hernia repair has lagged behind most other European countries with a similar healthcare system. This conservative attitude is also seen in other surgical fields such as colorectal and hepato-biliary surgery. Until recently, the proportion of inguinal hernia repairs performed with minimally invasive surgery has also been low in Sweden, where a more widespread adoption of the TEP and TAPP techniques occurred. Robotic assisted ventral hernia repair was not introduced as a routine procedure in ventral hernia repair in Sweden during this time.

The aim of this eleven-year nationwide population-based cohort study was to explore the impact of surgical approach on outcome of ventral hernia, and differences in serious complication rates between primary and incisional hernia.

### Hypothesis:

The risk for a serious surgical complication is higher after laparoscopic ventral hernia repair (LVHR) than after open ventral hernia repair, and higher after incisional than after primary hernia repair.

## Material and method

### Material and methods

Patient data on individuals who had a ventral hernia repair between 2004 and 2014 were retrieved from the National Patient Register kept by the Swedish National Board of Health and Welfare. The Swedish National Inpatient Register (IPR), also called the Hospital Discharge Register, was established in 1964. The IPR has complete national coverage since 1987 and is part of the National Patient Register. Currently, more than 99% of all somatic and psychiatric hospital discharges are registered in the IPR. Diagnoses in the IPR are coded according to the Swedish version of the international classification of disease (ICD) system, first introduced in 1964 (adapted from the WHO ICD classification system). It is mandatory for all physicians, private or publicly funded, to deliver data to the IPR. Since 2001, procedures performed at ambulatory surgery units are also included in the register. A review performed in 2011 showed its validity to be high for patients with severe disease and causal complications, and procedures with missing data were less than 1% [12].

This register provides the opportunity to analyse the risk for occurrence of adverse events during and after ventral hernia repair in routine clinical practice. Patients with an ICD-code indicating ventral hernia repair were gathered (JAD, JAF, JAE and JAG) with sub groups, and codes for cardiovascular complications, reoperation, admission to intensive care unit (ICU), and death within 30 days after index surgery were included in the final dataset. Inclusion of all ventral hernia procedure codes enabled comparison of open and laparoscopic ventral hernia repairs.

Patients below 18 years of age were not included in this material. Patients with concomitant bowel surgery are excluded from the data set since this is a group that might differ considerable from ventral hernia repairs without bowel surgery in terms of morbidity and mortality. This means that patients with acute presentation that require bowel resection are also excluded from analysis. The majority of ventral hernia repairs in Sweden were during the studied period performed by generalists. Ventral hernias are managed at all different kind of hospitals: rural, private and University settings. Data were dichotomised into open and laparoscopic surgery and stratified for primary and incisional hernias. A list of variables is supplied in the Appendix. The study was conducted according to the STROBE checklist.

The Regional Ethics Review Board in Umeå (2017–205/32) approved the study.

### Statistical methods

Patient data were acquired from the Patient Register as an Excel® file (Microsoft Corp.). An accredited statistician at Umeå University performed database processing in R®. Final statistical analyses of the data were performed using Statistica version 12 (Statsoft, Tulsa, OK, USA) and IBM SPSS Statistics 26 (SPSS Inc.). Statistical significance was tested by Chi-square analysis where a p value < 0.05 was regarded as significant. Uni- and multivariable analyses were performed in Statistica on models where reoperation during index admission, mortality, and incisional hernia respectively served as dependent variables. Age, sex, and laparoscopy served as independent variables.

Logistic regression was used for uni- and multivariable analyses. Variables not significant in the univariable analyses were excluded from the multivariable analysis, and histograms of risk for reoperation and risk of death for different age groups were performed in Excel.

During the work process, data were handled according to the guidelines stated by SAMPL.

## Results

**Fig. 1:**
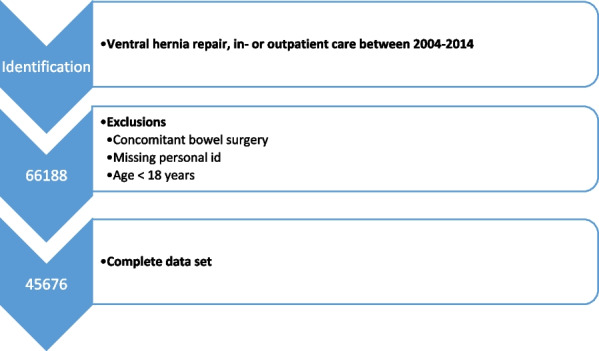
Flowchart of patients included in the analysis

Using predefined criteria, the dataset comprised 66,188 admissions. Hernia repairs with concomitant bowel surgery were excluded, leaving 45,676 admissions between 2004 and 2014 for analysis (Fig. 1). Patients were between 18 and 101 years of age. The majority of repairs were open 97.3% (44,444) leaving 2.7% (1232) performed laparoscopically. There were no obvious differences between open and laparoscopic surgery in terms of death, and risk for major medical or surgical complication (Table 1).

**Table 1:** open and laparoscopic ventral hernia repair and complications

	Open (%)	Laparoscopic (%)
Reoperation, index admission	277 (0.6)	6 (0.5)
Surgical complication	81 (0.2)	1 (0.1)
Medical complication	80 (0.2)	4 (0.3)
Reoperation/Readmission	92 (0.2)	5 (0.4)
Death within 30 days	40 (0.1)	3 (0.2)
Total	44 445	1231

Of the total cohort, 36% (16,670) were incisional hernias and 64% (29,006) primary hernias. Forty-six per cent (21,141) of all repairs and 55% of laparoscopic repairs were on women. No apparent differences were seen between primary and incisional hernias regarding major medical or surgical complications (Table 2). Mean age was 54 years for open repair and 56 years for laparoscopy. The proportion of patients over 70 years was 16% for open repair and 18% for laparoscopy. Of repairs conducted with laparoscopy, 61% were for incisional hernia while 34% of open repairs were for incisional hernia.

**Table 2:** Open versus laparoscopic repair with subgroup analysis of incisional hernia and primary hernia repair regarding reoperation, death, and surgical complication

	Open surgery (%)			Laparoscopic (%)		
	Incisional	Primary	P	Incisional	Primary	P
Reoperation, index	154 (1)	123 (0.4)	0.001	5 (0,7)	1 (0.2)	0.26
	154 (1)			5 (0.7)		0.41
		123 (0.4)			1 (0.2)	0.46
Surgical complication	53 (0.3)	28 (0.1)	0.001	1 (0.1)	0 (0.0)	0.42
Medical complication	51 (0.3)	29 (0.1)		2 (0.3)	2 (0.4)	
Reoperation/readmission	53 (0.3)	39 (0.1)		4 (0.5)	1 (0.2)	
Death within 30 days	26 (0.2)	14 (0)	0.001	2 (0.3)	1 (0.2)	0.84
	26 (0.2)			2 (0.3)		0.5
		14 (0)			1(0.2)	0.13
Total	15,919	28,526		751	480	

**Fig. 2:**
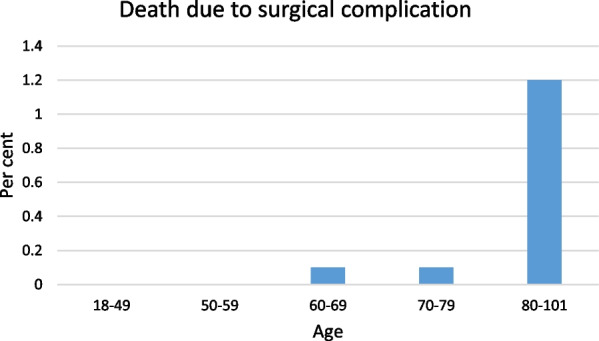
Risk of death correlated to age

Forty-three patients (0.01%) died from complications within 30 days after index surgery. Age was the most important risk factor for death after repair, and 88% (38/43) of patients who died were older than 60 years (Fig. 2). Age (continuous variable, univariate analysis) was a risk factor for death after both incisional and primary hernia repair, OR 1.13 (1.09–1.18 p < 0.001) and OR, 1.14 (1.09–1.19 p < 0.001) respectively.

In uni- and multivariable analyses, age was a risk factor for reoperation in primary hernia repair: OR 1.06 (1.05–1.08 p < 0.001) and 1.06 (1.05–1.07 p < 0.001) respectively.

Female sex was a risk factor for reoperation in both uni- and multivariable analyses: OR 1.63 (1.14–2.32), and OR 1.84 (1.29–2.62 p < 0.001) respectively (Table 3).

**Table 3:** Uni- and multivariable analysis, reoperation primary hernia

	Univariable		Multivariable	
	Odds ratio	P	Odds ratio	P
Age	1.06 (1.05–1.08)	< 0.01	1.06 (1.05–1.07)	< 0.01
Female	1.63 (1.14–2.32)	0.01	1.84 (1.29–2.62)	< 0.01
Laparoscopy	0.48 (0.07–3.46)	0.47		

Gender was not a risk factor for mortality or reoperation after incisional hernia. Age was a risk factor for reoperation after incisional hernia repair OR 1.02 (1.00–1.03, p = 0.01).

**Fig. 3:**
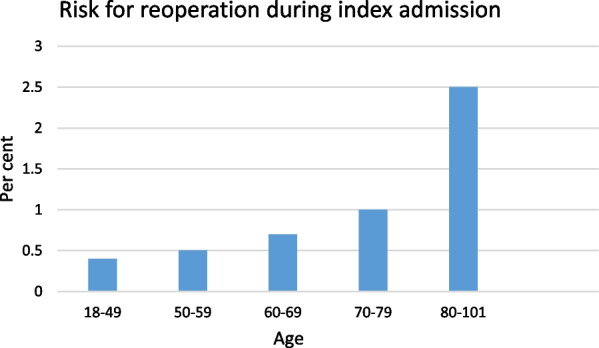
Risk for reoperation correlated to age

Patients aged 80 years or more had a 2.5-times higher relative risk for complication leading to reoperation, and a 12-times higher relative risk of death than patients aged 70–79 years (Figs. 2, 3).

Fifteen of forty-three deaths occurred after primary repair. Of these, thirteen were older than 60 and eight older than 80 years.

There were 4965 (11%) reoperations for recurrent hernia during the study period (Table 4). Basic demographic variables are seen in Table 5.

**Table 4:** Number of patients with multiple hernia operations

Number of interventions	Patients
2	2133
3	200
4	20
5	0
6	2
7	1
	4965

**Table 5:** Basic demographic data

Patient characteristics	N
Age (mean) (range)	54 (18–101)
Female	21 141 (46%)
Incisional hernia	16 670 (36%)

Table 6 show suture and mesh repair of incisional and primary hernias and the risk of reoperation, complication and mortality.

**Table 6:** Suture and mesh repair of incisional and primary hernias and the risk of reoperation, complication and mortality

	Incisional hernia (%)		P value	Primary hernia (%)		P value
	Suture	Mesh		Suture	Mesh	
Reoperation index admission	51 (1.1)	73 (0.3)	< 0.001	36 (1.2)	123 (0.9)	0.173
Surgical complication	10 (0.2)	18 (0.1)	0.007	9 (0.3)	45 (0.3)	0.732
Medical complication	4 (0.1)	27 (0.1)	0.575	9 (0.3)	44 (0.3)	0.780
Reoperation, readmission	19 (0.4)	21 (0.1)	< 0.001	12 (0.4)	45 (0.3)	0.615
Death within 30 d	7 (0.1)	8 (0.0)	0.002	3 (0.1)	25 (0.2)	0.290
Total	4831	24,175		3079	13,591	

Table 7 show the proportion of suture and mesh repair in primary and incisional ventral hernia repair.

**Table 7:** Proportion of suture and mesh repair in primary and incisional ventral hernia repair

	Suture (%)	Mesh (%)	P value
Incisional hernia	4831 (16.7)	24,175 (83.3)	< 0.001
Primary hernia	3079 (18.5)	13,591 (81.5)	

## Discussion

Both open and laparoscopic approaches appear to be safe for ventral hernia repair, with low risk for reoperation and low mortality. This finding is particularly valid for open hernia repair since most are performed via an open approach in Sweden.

The considerable difference in numbers between open and laparoscopic procedures performed limited our ability to display superiority of either method. It is important to realise that there is a difference between data generated from prospective trials and those derived from a national register that represents procedures performed in every day practice at a national level.

The risk of death dramatically increases in the group over 80 years after both primary and incisional hernia repair. This calls for precautionary measures, even in technically simple cases.

Numerous variables need to be considered when evaluating the indication for ventral hernia repair. A “watchful waiting” strategy avoids an unnecessary procedure, but has the disadvantage that the hernia aperture might enlarge while waiting, making repair more complicated as well as increasing loss of domain [13]. Furthermore, acute surgery is associated with higher morbidity and mortality.

The outcome of ventral hernia repair depends to some extent on gender [14, 15]. There was a higher risk for reoperation during index admission in women with a primary hernia. The reason for this is unclear and needs further analysis in a prospective setting with hernia-specific variables. Missed hernia, probably due to multiple defects in women with diastasis recti, has been described as a reason for reoperation [16]. Women are also over-represented in injury claims after ventral hernia repair [16]. Previously published papers claim that ventral hernia repair in women has less favourable outcome regarding surgical site infection [17], readmission [18], and chronic pain [19]. The impact of gender in ventral hernia repair is clearly a complex matter. Differences in fat distribution between men and women, a higher proportion of incisional hernias among women (due to caesarean and gynaecological procedures) are possible contributing factors [15]. Furthermore, alterations in hormonal balance during pregnancy affects collagen composition. The midline of the abdominal wall weakens as the components of the extracellular matrix change due to alteration in the progesterone-oestradiol balance and release of corticosteroids. In addition, increased concentrations of the hormone relaxin directly stimulate metalloproteinases that degrade the matrix [20].

Laparoscopically operated patients had a lower risk for acute reoperation during index admission compared to patients operated with an open technique. The lower rate of reoperation could indicate that minimally invasive techniques have lower complication rates. However, bias due to case-mix may play a role here since complicated cases are not always considered eligible for laparoscopy.

Formal conclusions regarding differences between open and laparoscopic hernia repair are difficult to draw from the dataset in the present study. But since there was a higher proportion of incisional hernias in the laparoscopy group and age groups were equally distributed, there was no obvious pattern in the analyses that could explain the lower complication rate in the laparoscopy group in terms of less complex cases or younger healthier patients.

Previously reported data from Swedish and Finnish national patient insurance companies suggest that the risk for inadvertent enterotomy is higher in laparoscopically operated patients [16, 21]. The data presented here, from a much larger cohort, do not seem to support this.

The risk for reoperation and death was somewhat higher after incisional than after primary hernia repair for both open and laparoscopic repairs. This finding is in in line with previously presented data [5, 22]. The fact that an incisional hernia often has a wider aperture than a primary hernia is an important factor contributing to poorer outcome after incisional hernia repair [22].

Increased risk of reoperations after incisional hernia repair was also confirmed in a systematic review of the literature and meta-analysis published in 2019. In that analysis, incisional hernias had an increased risk of recurrence, longer length of stay and longer operative time compared to primary ventral hernia repair. Other complications did not differ significantly between primary and incisional hernias in that meta-analysis, which is a finding that is also in line with the results from the present paper [6].

The proportion of suture repair of incisional hernias in this study was surprisingly high. A cohort displaying recent years might show different proportions of repairs. During the studied period, the results from the randomised study by Luijendijk et al. [23] had not completely been implemented in ventral hernia repair in Sweden. The fact that suture repair in incisional hernias seems associated with increased risk of mortality is interesting but can be a result of selection bias since patients with the highest preoperative risk might be selected to a suture repair to limit the surgical trauma. Mesh repair does not seem to significantly increase risk of reoperation or mortality.

Suture repair of incisional hernias was associated with an increased risk of reoperation and should be abandoned.

Patient frailty is a factor to consider when planning surgery [24, 25]. Preoperative optimisation, evaluation of indication, and method chosen for repair are variables influencing morbidity and mortality [26]. The proportion of incisional hernia patients that are frail is higher than among those with a primary hernia.

Nevertheless, we found that age is also a risk factor for mortality after repair of primary hernia, a procedure that is usually technically uncomplicated and the aperture modest.

The findings of the present study indicate that age is the most important risk factor for death. Clinicians should take note and perhaps introduce more stringent indication criteria for repair in different age groups, and that a “watchful waiting” strategy should be more widely applied in the elderly patient group [27].

Mortality and major adverse event rates in the present material could be considered surprisingly low. The fact that patients who had concomitant bowel surgery were excluded from the analysis probably contributed to this. The exclusion also remove patients with acute presentation in need of a bowel resection from the analysis. This is a group with considerably increased risk of complication and mortality [28]. Concomitant bowel surgery during ventral hernia repair probably increases the risk for reoperation, sepsis, and infection [29, 30]. There are also reports indicating that the risk for anastomotic leakage in bowel surgery can be increased by concomitant incisional hernia repair [31]. This is an issue that needs further research to evaluate and understand the underlying pathophysiology involved. Large hernia aperture is an important risk factor for serious complications in ventral hernia repair [13, 22]. Hernia aperture size is routinely registered in specialised quality registers but not in larger registers with a wider focus as used in this study. Though data on size were lacking, most hernias described in this material were probably small.

Analyses of surgical procedures using register data should be regarded with some degree of scepsis [32]. One presumption is that not all patients undergoing ventral hernia repair are eligible for laparoscopic surgery, and that methods are not interchangeable. Our ability to draw conclusions from register data was limited since we could only analyse the variables that currently exist in the register. Comorbidities, choice of mesh, method of fixation, and other variables of interest in hernia research were not available in our database. Randomised controlled trials reflect a controlled situation over a limited time, while epidemiology data reflect routine clinical practice on a population basis. These two sources of information complement one another.

The main strength of the present study is the large study cohort with more than 99% completeness The positive predictive value well over 90% also indicates accuracy of allocation of patients to the study groups. The weakness of the present study is the lack of data on comorbidity and hernia-specific variables.

The low number of surgical complications registered may be a sign of high quality, but it may also be due to poor reporting. Reoperation and death are solid variables in Swedish national registers, which is why these two variables were chosen as key outcomes [33]. The overall low mortality and reoperation rates are interesting per se but difficult to compare with existing randomised trials and other published register data. Although data on hernia recurrence were not available for analysis, we did have the number of patients undergoing reoperation after hernia repair during the study period. The 11% reoperation rate in the present study is in line with previously reported data and can thus be an indication of external validity [34].

## Data Availability

The data and material can be shared upon reasonable request but have to be approved by the ethical authority in Sweden. To request the data from this study please contact the first author at mikael.e.lindmark@umu.se.
